# Investigating a Devonian vertebrate assemblage with synchrotron microtomography of coprolites

**DOI:** 10.1038/s41598-026-63718-2

**Published:** 2026-08-10

**Authors:** Arnaud Rebillard, Vincent Dupret, Pavel Beznosov, Per Erik Ahlberg

**Affiliations:** 1 Museum Für Naturkunde Leibniz-Institut Für Evolutions- Und Biodiversitätsforschung, Invalidenstraße 43, 10115 Berlin, Germany; 2 Humboldt-Universität Zu Berlin, Institut Für Biologie, Invalidenstraße 42, 10115 Berlin, Germany; 3 Département Origines & Evolution (CP 38), UMR 7207 Centre de Recherche en Paléontologie - Paris (CNRS-MNHN-Sorbonne Université), Muséum National d’Histoire Naturelle, 8 Rue Buffon, 75231 Paris Cedex 05, France; 4 Department of Organismal Biology, Uppsala University, Norbyvägen 18A, 752 36 Uppsala, Sweden; 5 Institute of Geology, FRC Komi Science Centre, UB of RAS, 54 Pervomayskaya St., Syktyvkar, 167982 Komi Republic Russian Federation

**Keywords:** Sarcopterygii, Acanthodii, Dipnoi, Trophic web, Bromalites, Ecology, Ecology, Evolution, Zoology

## Abstract

Fossilised faeces (coprolites) are a unique source of information as they record palaeontological evidence of animal behaviour (autecology) as well as interactions between extinct organisms and their environment (synecology). Four samples bearing coprolites found in a newly discovered vertebrate locality in the Kumushka Formation dated to the Givetian-Frasnian transitional interval of the Devonian sequence of North Timan (NE Europe) were scanned using propagation phase-contrast synchrotron microtomography. A total of seven coprolites were recovered and segmented to digitally extract their entire internal contents. The three-dimensional preservation of the inclusions allowed for their anatomical and taxonomical identification. Acanthodian and fleurantiid-like dipnoan remains are abundantly found in every sample, along with fewer holoptychiid sarcopterygian elements. The contents of the coprolites indicates an opportunistic feeding behaviour, with remains from multiple trophic levels present across the different samples. The size of some remains reveals feeding on juveniles. Sarcopterygians *Eusthenopteron, Laccognathus* and *Glyptolepis* are inferred to be potential producers of the coprolites based on their sizes, morphologies, inclusions and their internal architecture. The taxa preserved within the studied coprolites and from body fossil remains, are placed in a trophic pyramid, providing evidence for the early establishment of a complex multi-level trophic chain at the Givetian-Frasnian transition.

**Supplementary Information:**

The online version contains supplementary material available at 10.1038/s41598-026-63718-2.

## Introduction

The Late Devonian (383–359 million years ago) was a key epoch in the evolution of non-marine ecosystems: tetrapods became widely distributed while land plants and arthropods diversified. Intensive research has been conducted for nearly a century^1–6^ but these works focused mainly on phylogeny and morphology rather than on ecosystems. Coprolites, i.e., fossilised faeces, offer valuable insights into palaeoenvironments and allow to better understand trophic networks^7^ and faunal diversity^8^.

During the Devonian Period, coprolites are increasingly common and are known from a range of depositional environments spanning marine to fully non-marine environments^9^. Many well documented examples derive from marginal marine or shallow epicontinental basins, including the Escuminac Formation (Canada)^10,11^, the Pimenteira Formation (Brazil)^12^ and the Lode assemblage (Latvia)^11,13^. Coprolites from non-marine assemblages are particularly known from the Old Red Sandstone continental basins of Scotland, such as Achanarras^14,15^ Tillywhandland^16^ and Duntrune^9^.

The studied material comprises four coprolite-bearing rock samples collected in 2017 from the Devonian deposits of North Timan (Nenets Autonomous District, Russian Federation) by one of the authors (P.B.). The specimens were obtained from a newly discovered and still poorly described Lagerstätte^17^. Geologically, the Timan Ridge is a peneplanated highland that forms the northeastern margin of the East European Platform. In its northern part, Devonian sedimentary and effusive rocks are predominantly exposed in the axial zone of the ridge. This area was affected by tectonic and volcanic activity during the Middle to Late Devonian transition. The sequence includes three^18^ to eight^19^ basalt flows interbedded with siltstone and sandstone members which together constitute the Kumushka Formation^20^. The stratigraphic level of this site corresponds to the upper part of the Timanian Regional Stage^21^, which has been tentatively correlated to the lowermost part of the Frasnian^22^.

**Fig. 1:**
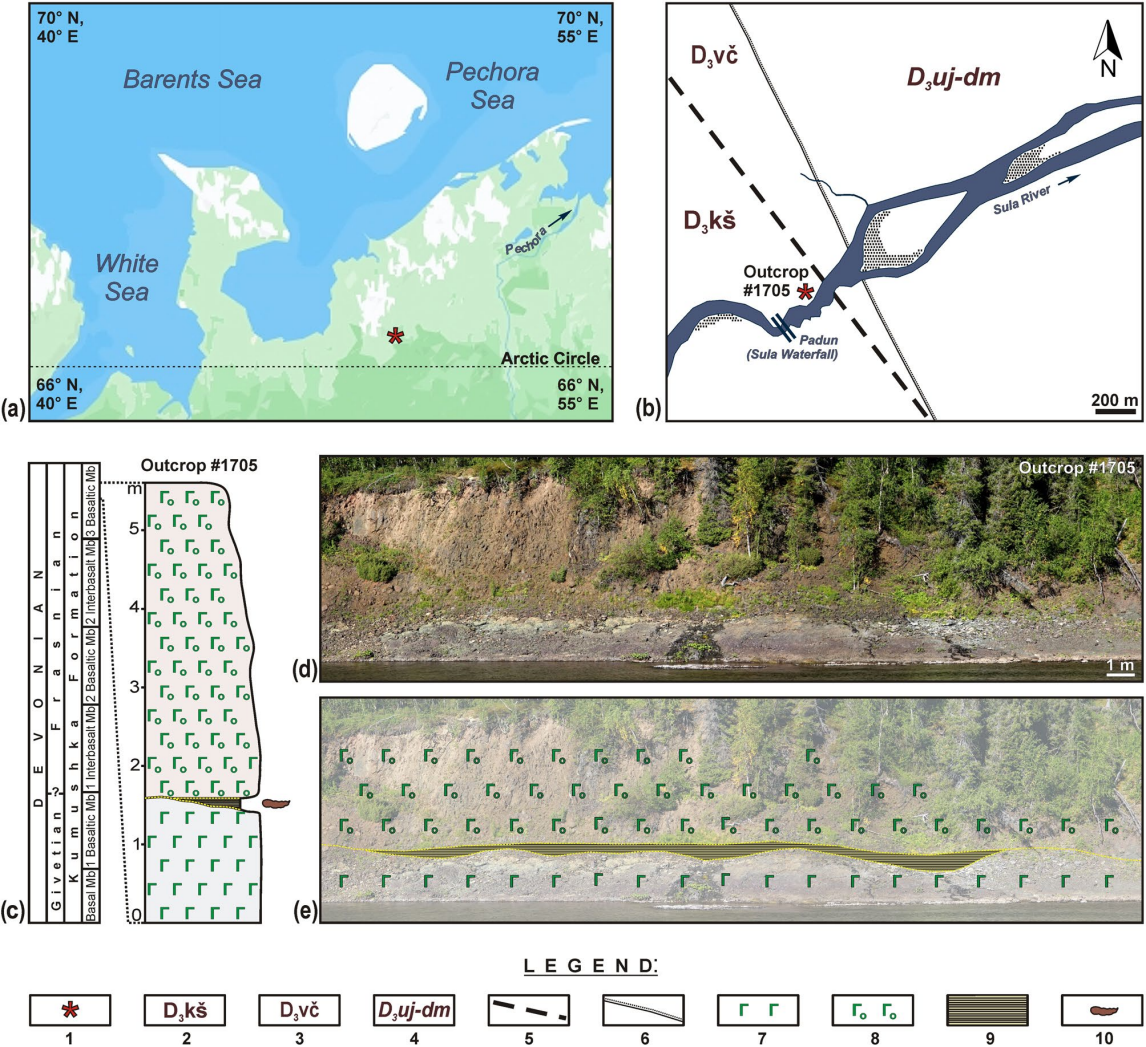
Geographical (**A**-**B**) and geological (**C**-**E**) positions of the studied coprolite site. 1 — location of the Outcrop #1705; 2 — area of the Kumushka Formation outcrops; 3 — area of the Vyucheyskiy Formation outcrops; 4 — area of outcrops of sandy deposits of the Ust’yaregian – Domanikian regional stages; 5 — fault; 6 — synchronous stratigraphic boundary; 7 — basalt; 8 — amygdaloidal basalt; 9 — laminated terrigenous rocks of the coprolite-bearing inter-basalt lens; 10 — coprolites. Regional Stratigraphic Chart fragment compiled from^18,20,21^: Photograph by P.B.

The Devonian sequence of the North Timan yields abundant and diverse vertebrate body fossils^23,24^. Most of these localities belong to the Upper Devonian supra-basalt terrigenous deposits, formed in a wide delta plain environment^25^. The outcrops underlying the Kumushka Formation are poorly characterised palaeontologically^26^. A new unique fossil site was recently discovered on the left bank of the Sula River, 0.1 km downstream of the Sula Waterfall (Fig. 1). Here, within the third (upper) basalt member of the Kumushka Formation, a bone-bearing lens characterised by laminated terrigenous rocks was found among the massive and spheroidal lavas. The lower boundary of the lens is clearly visible from the overlap of basalts onto the thin horizontally laminated siltstones and coaly shales. The sedimentary rocks of its upper part are thermally altered, chloritised and tightly baked against the lower surface of the overlying basalts. The observable length of the lens is about 50 m, the thickness reaches 0.8 m in places, but in most parts does not exceed 0.2–0.3 m. These fine-grained laminated sediments are indicative of deposition in a low energy conditions, suggesting formation in a lacustrine environment of a short-lived lake within a volcanic landscape.

The lens yields abundant and well-preserved body fossils representing about ten fish taxa, including the sarcopterygians *Laccognathus* sp., *Glyptolepis* sp., cf. *Eusthenopteron* sp., Osteolepiformes gen. indet., Dipnoi gen. indet., acanthodians *Acanthodes*? sp., cf. *Haplacanthus* sp., cf. *Nostolepis* sp., and placoderms *Asterolepis radiata* and *Bothriolepis*? sp. Some specimens are represented by articulated and semi-articulated skulls and other skeletal fragments. In addition to vertebrate body fossils, the rocks of the inter-basalt lens also include plant remains of the lycophyte *Helenia karakubensis* and the progymnosperm *Callixylon trifilievii* (Snigirevsky, pers. comm.), as well as numerous coprolites, the latter being the subject of this study.

The coprolites have varied shape and size (see Table 1), which reflect the diversity of the aforementioned ichthyofaunal community. The Sula River fossil site is currently the only known locality of such abundant and well-preserved Devonian coprolites in Timan.

**Table 1:** List of analysed specimens and coprolites including their size, spirality, morphology and inclusions.

Specimen	Coprolite	Size (in mm)	Spiral of non-spiral ?	Morphology	Morphotype	Inclusions
#IGKSC 324/19	#IGKSC 324/19	Length: 19,3 Width: 8,55	Spiral-whorls undefined	Ovoid elongated	Morphotype 1	Holoptychiid scale fragments, numerous and diverse fleurantiid-like dipnoan skeletal remains, possible dipnoan vertebra, acanthodian scales
#IGKSC 324/59	#IGKSC 324/59A	Length: 23,1 Width: 8,7	Spiral whorls undefined	Ovoid elongated	Morphotype 1	Diverse fleurantiid-like dipnoan remains including tooth-plates, osteichthyan scales, diplacanthiform acanthodian fin spine, acanthodid-like scapulocoracoid, acanthodian scales, various indeterminable acanthodian remains
#IGKSC 324/59	#IGKSC 324/59B	Length: 17,4 Width: 9,1	Non-spiral	Fraction of a cylinder shape	///	Holoptychiid teeth (coronoid tooth, marginal teeth and a parasymphysial tooth), fleurantiid-like dipnoan prearticular tooth-plate and lepidotrichia, sarcopterygian fangs, osteichthyan and acanthodian scales
#IGKSC 324/59	#IGKSC 324/59C	Length: 7,1 Width: 6,6	Non-spiral	Flattened and rounded shape	///	Possible dipnoan clavicle, acanthodian scales
#IGKSC 324/25b	#IGKSC 324/25b	Length: 10 ,6 Width: 8,2	Non-spiral	Arrowhead shape	Morphotype 2	Fleurantiid-like dipnoan lepidotrichia, dipnoan tooth-plate and cranial rib, osteichthyan infraorbital bone, acanthodian scales
#IGKSC 324/31	#IGKSC 324/31A	Length: 8,8 Width: 7,1	Non-spiral	Arrowhead shape	Morphotype 2	Fleurantiid-like dipnoan indet. clavicle and unidentified elements, osteichthyan lepidotrichia, acanthodian scales
#IGKSC 324/31	#IGKSC 324/31B	Length: 8,1 Width: 7,8	Non-spiral	Arrowhead shape	Morphotype 2	Fleurantiid-like dipnoan supraneural shafts and a single lepidotrichium

Preliminary investigation of the coprolites by SEM and thin sections revealed contents including fish scales, possible soft tissue and parasitic cysts^27^ which confirmed the importance of this material, but also highlighted the importance of studying them by means of a non-destructive methodology. Subsequently, these coprolites underwent microtomographic scanning at the European Synchrotron Radiation Facility (ESRF) in Grenoble, France. For this study, we used 3D imaging techniques on these high resolution scans to virtually dissect the coprolites and generated 3D models to identify as many elements as possible. This study contributes to the description of this faunal assemblage, previously based on non-coprolite contents such as tooth and bony remains. This approach enables also the reconstruction of the ecosystem and trophic network of the Sula River locality, and refines taxonomic affiliations of remains recovered from the site.

## Results

As evidenced by the EDS analysis (see Supp file 1), the coprolite groundmass has heterogenous elemental composition and is mainly composed of P_2_O_5_, CaO and SiO_2_, in contrast to the host sediment, primarily composed of SiO_2_ and Al_2_O_3_ but depleted in P_2_O_5_ and CaO. The large amount of phosphate in the coprolitic groundmass probably derives from the dissolved skeletal and soft tissues from digested prey. Such phosphate accumulation likely favoured mineralisation induced by bacterial activity, which played an important role in the preservation and fossilisation of the faeces and the inclusions they contain^28–31^. The bacterial activity is also evidenced by the presence of framboidal pyrite, which occurs both inside the coprolites and externally within the surrounding rock matrix and plant remains^32^.

**Fig. 2:**
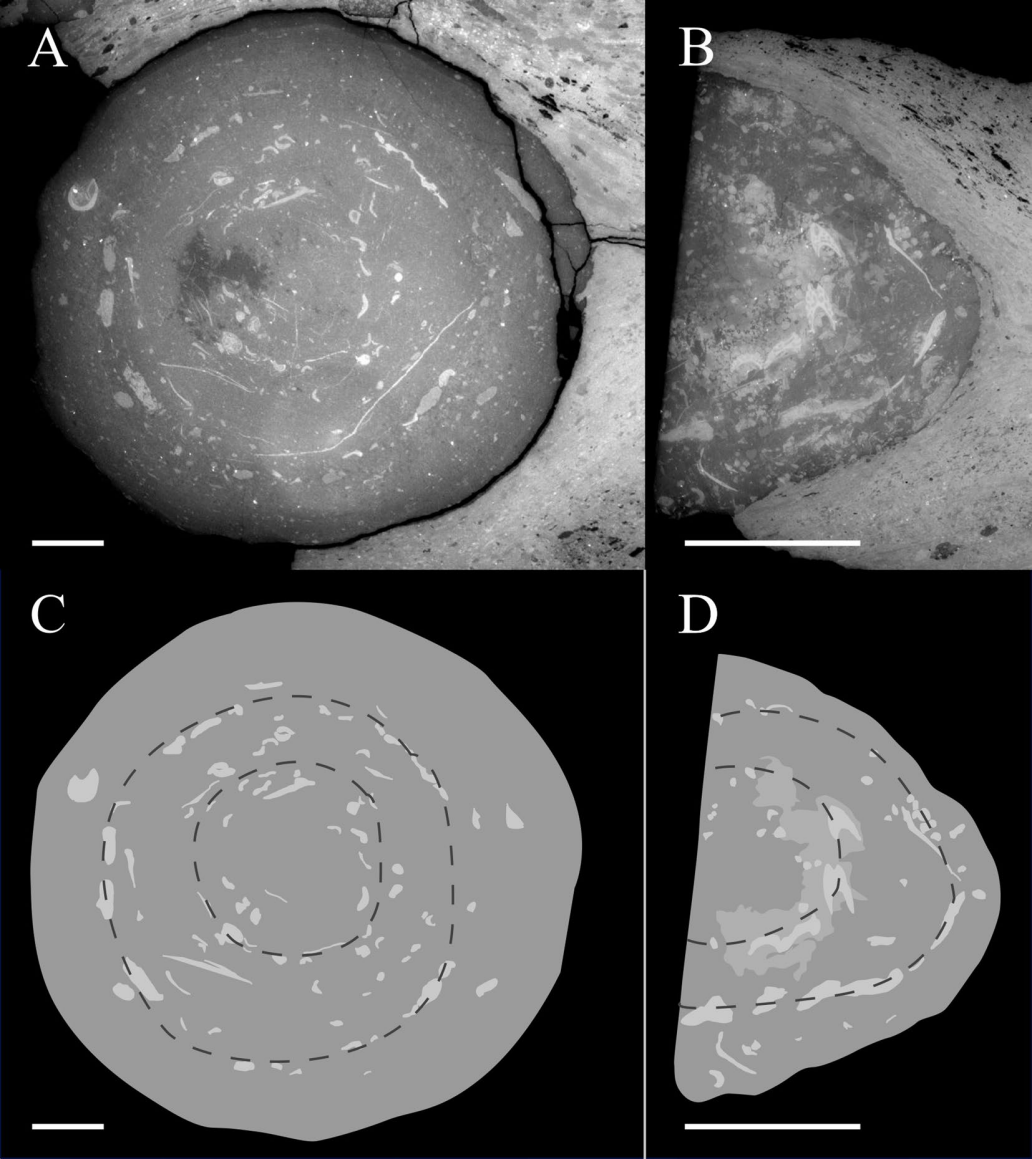
Synchrotron tomographic slices of coprolites #IGKSC 324/19 (**A**) and #IGKSC 24/59A (**B**) with corresponding interpretative drawing (**C**-**D**) highlighting the spiral architecture of the inclusions, along the dotted circles. Scale bars: 5 mm.

**Fig. 3:**
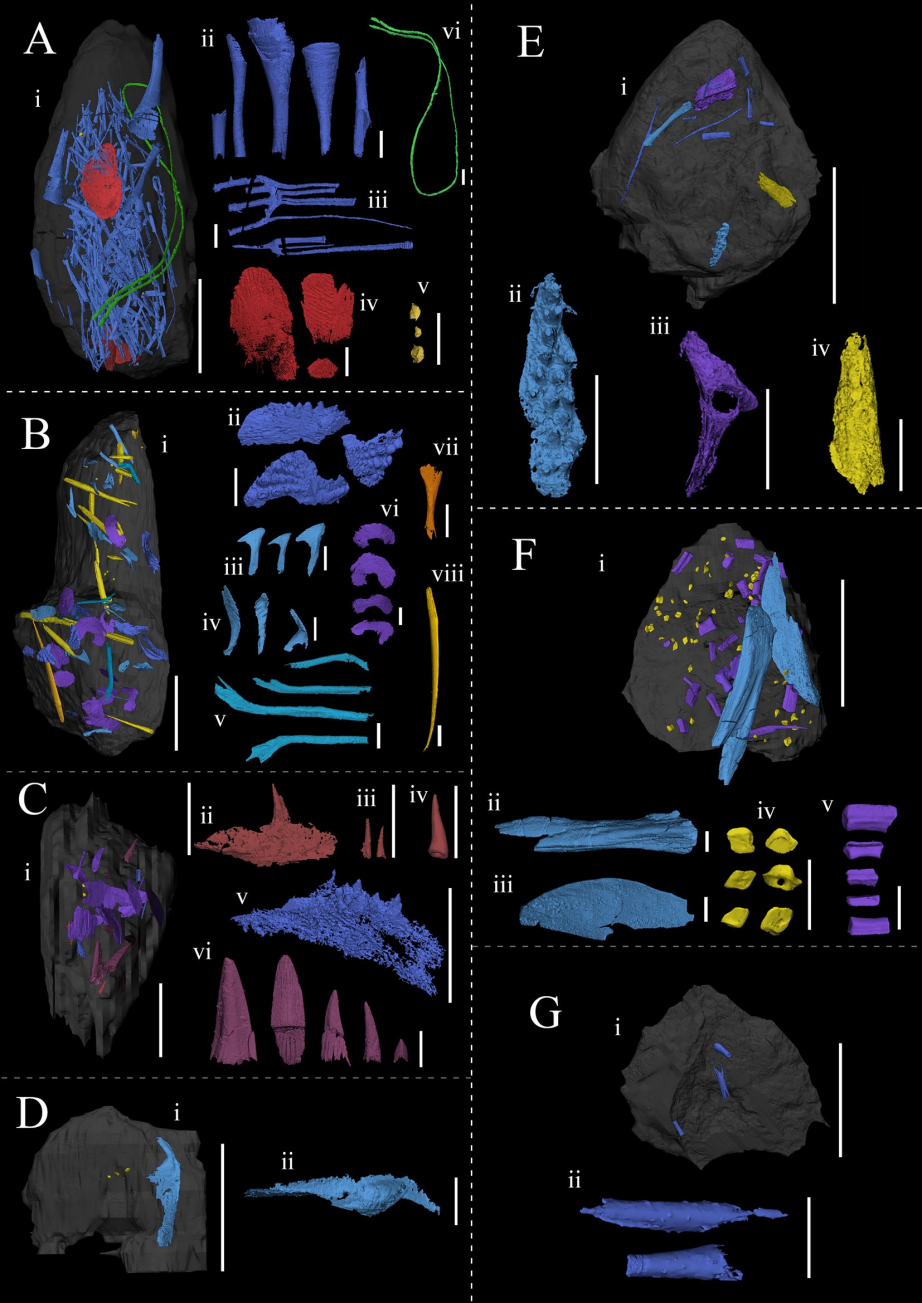
3D reconstructions of the studied coprolites, rendered in semi-transparency, and a selection of their contents (Sula fossil site, Nenets Autonomous Okrug, Arkhangelsk Region, RF, Kumushka Formation, Givetian-Frasnian transition beds). **A**: coprolite #IGKSC 324/19 with dipnoan supraneural fragments (A_ii_) and articulated lepidotrichia (A_iii_), sarcopterygian scale fragments (a *Glyptolepis*-like holoptychiid and a possible dipnoan) (A_iv_), acanthodian scales (A_v_) and plant remain (A_vi_); **B**: coprolite #IGKSC 324/59A with fleurantiid-like dipnoan prearticular toothplates (B_ii_), dipnoan. skull bones (B_iii_), shoulder girdle elements (B_iv_) and ribs (B_v_), osteichthyan scale fragments (B_vi_), acanthodid-like acanthodian scapulocoracoid (B_vii_), and diplacanthiform-like acanthodian fin spine (B_viii_); **C**: coprolite #IGKSC 324/59B with holoptychiid coronoid (C_ii_), marginal (C_iii_) and parasymphysial (C_iv_) teeth, fleurantiid-like dipnoan prearticular toothplates (C_v_), sarcopterygian fangs (C_vi_) and acanthodian scales (C_vii_); **D**: coprolite #IGKSC 324/59C with a dipnoan cleithrum (D_ii_) and acanthodian scales (D_iii_); **E**: coprolite #IGKSC 324/25b with a fleurantiid-like dipnoan tooth plate (E_ii_), osteichthyan infraorbital bone (E_iii_), and a possible acanthodian fin spine (E_iv_); **F**: coprolite #IGKSC 324/31A with a dipnoan unidentified element (F_ii_), and clavicle (F_iii_), acanthodian scales (F_iv_), and osteichthyan lepidotrichia (F_v_); **G**: coprolite #IGKSC 324/31B with fleurantiid-like dipnoan supraneural shaft fragments (G_ii_). Scale bars: 5 mm ( A_i_, B_i_, C_i_, D_i_, E_i_, F_i_, G_i_ ) ; 1 mm (A_ii-v_, B_ii-viii_, C_ii-vi_, D_ii-iii_, E_ii-iv_, F_ii-v_, G_ii_).

The studied coprolites exhibit a variety of morphologies (Fig S1-S4). Specimens #IGKSC 324/19 and #IGKSC 324/59A are elongated ovoid with one rounded end and the other slightly tapering. Specimens #IGKSC 324/25b, #IGKSC 324/31A and #IGKSC 324/31B have a flattened rounded arrow-head like shape (characterised by a small length-to-width ratio, one tapering end, and one wider rounded end), and are slightly concavo-convex. Specimen #IGKSC 324/59B appears to be a fragment with a sub-cylindrical shape and poorly-defined edges. Specimen #IGKSC 324/59C has a flattened and rounded shape. Specimens #IGKSC 324/59A and #IGKSC 324/59B are incomplete, having been sectioned previously for cross-sectional study. None of the studied coprolites exhibits a visible external spirality. However, the skeletal inclusions in specimens #IGKSC 324/19 and #IGKSC 324/59A are spirally arranged along poorly defined whorls on the resulting synchrotron tomogram (Fig. 2). The spirality is also observable on the 3D renders (Fig. 3A_i_, B_i_,) and supplementary videos (Movies S1-S2). Despite the poor definition of whorls, it is highly unlikely that such spiralled internal architecture, appearing as concentring rings from a transverse tomogram, would be due to other types of process; Moreover, coprolite content is typically isolated from external processes, hence leading to exceptional preservation of the remains and their arrangement. The remaining coprolites lack such a clear spiral internal architecture (Fig. 3C-G). The size, morphology and content of each coprolite are summarised in Table 1.

The coprolites contain a large number of inclusions from different taxa, preserved in exceptional three dimensional detail, allowing us to gain an insight into the fauna that was preyed upon in the Sula River locality.

The most abundant and diverse remains are skeletal elements of fleurantiid-like dipnoans; prearticular tooth plates^33,34^ displaying several rows of conical teeth organised in radiating rows^35^(Fig. 3B_ii_,C_v_,E_ii_), hourglass-shaped supraneural bones (Fig. 3A_ii_,G_ii_) and many short isolated lepidotrichia, appearing thin and rather hollow in their central part. In addition, shoulder girdle elements (Fig. 3B_iv_,D_ii_,F_iii_), lightly ossified ribs (Fig. 3B_v_) and probable skull bones of a small, unidentified lungfish, were retrieved (Fig. 3B_iii_).

A few scales (Fig. 3A_iv_) were recovered from specimen #IGKSC 324/19. These scales can be attributed to a porolepiform (Porolepiformes) holoptychiid sarcopterygian, based on their ornamentation consisting of ridges organised into radiating rows. We assign them to *Glyptolepis* sp. as body fossils of this genus have also been recovered from the same locality. Additional porolepiform remains, including one coronoid (Fig. 3C_ii_), marginal teeth (Fig. 3C_iii_) and a parasymphysial tooth (Fig. 3C_iv_), were recovered in coprolite #IGKSC 324/59B.

Acanthodian scales (Fig. 3A_v_,F_iv_) are also extremely abundant. While only three are preserved within #IGKSC 324/19 and 52 in #IGKSC 324/31A, they are particularly numerous in the three coprolites from specimen #IGKSC 324/59, where thousands are clearly visible on each of the scans. Acanthodian fin spines were also found (Fig. 3B_viii_,E_iv_), one of them being extremely well preserved and attributed to the order Diplacanthiformes (Fig. 3B_viii_)^36^. Additionally, a potential scapulocoracoid was found, possibly belonging to a member of the family Acanthodidae^37^ (Fig. 3B_vii_).

Other content from the coprolites include indeterminate lungfish remains (Fig. 3F_ii_), sarcopterygian teeth displaying sharp cutting edges (Fig. 3C_vi_) and unidentified osteichthyan remains. Finally, a long, thin and tubular structure interpreted as a piece of plant remain was segmented (Fig. 3A_vi_). In the host sediment encasing #IGKSC 324/25b, a snout fragment of a large *Eusthenopteron*-like osteolepiform was found representing portions of the premaxilla and the vomer, with three fangs preserved in position (Fig. S5).

## Discussion

### Coprolite content

**Fig. 4:**
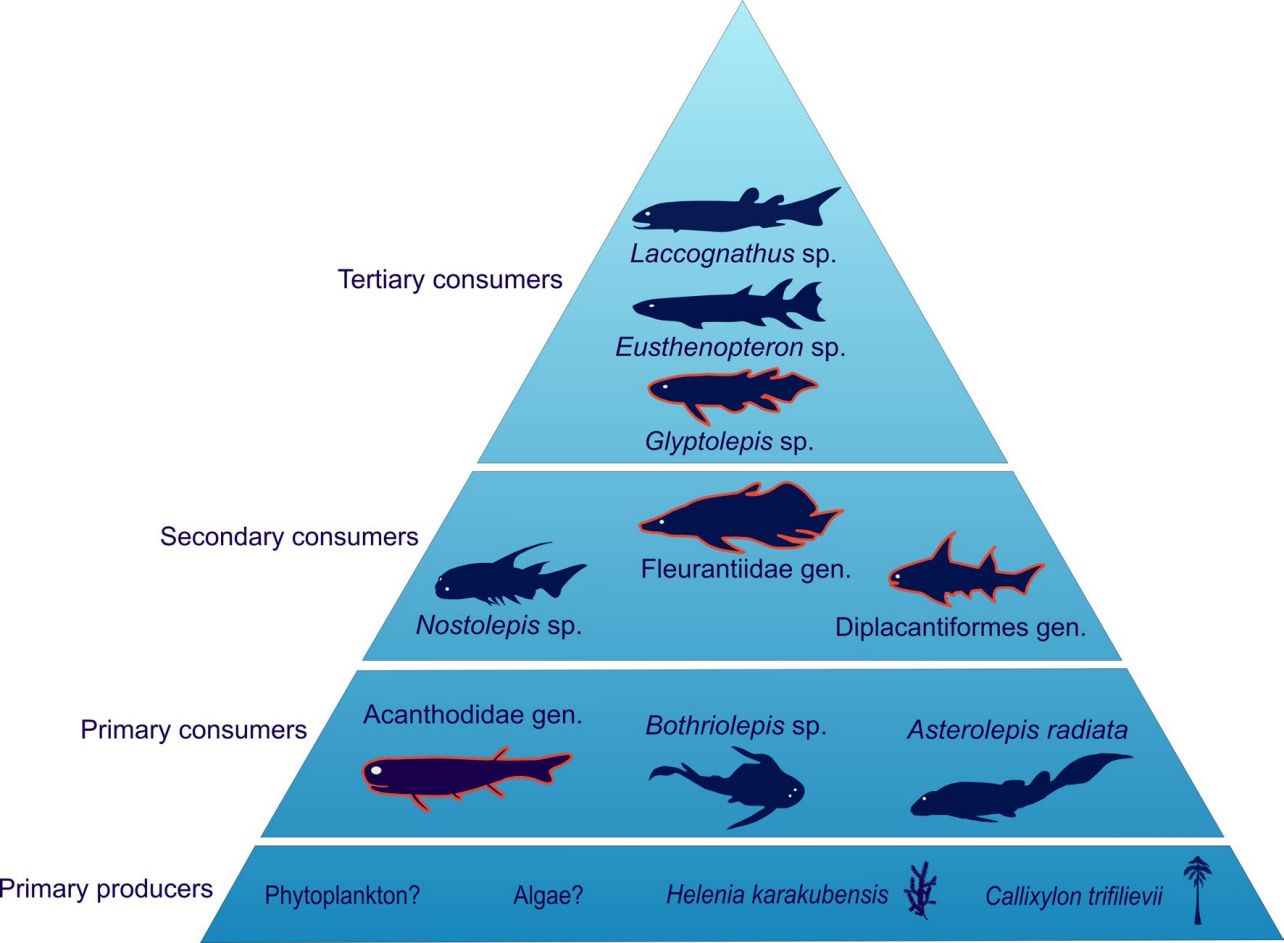
Trophic pyramid of the Sula site vertebrate assemblage, highlighting the importance of coprolites as “faunal aggregators”. Red outlines correspond to taxa preyed upon and recovered from the studied coprolites. The remaining faunal taxa were found as body fossils at the studied locality.

The studied coprolites show different morphologies and contain a rich diversity of faunal remains, suggesting a multi-level trophic web. The inclusions preserved in the coprolites provide direct evidence of the actual coexistence of the taxa identified within the fossilised faeces. The abundance of acanthodian remains in almost every coprolite could also suggest that the coprolites producers lived in a proximal (near-shore) environment^11,38,39^. Some dipnoan inclusions are notably smaller than those described in the literature for late Devonian specimens^40,41^, with shoulder girdle elements measuring less than 5 mm in length. This suggests they belong to juveniles, indicating the presence of a possible nearby nursery or spawning area. We treat acanthodids as primary consumers, since they are considered filter-feeding vertebrates^8,42,43^. Based on body size estimates, tooth size and morphology recovered from the coprolites and from comparison with existing literature, we classify fleurantiid dipnoans, *Nostolepis* sp. and diplacanthiforms as secondary consumers, and *Glyptolepis* sp. as a tertiary consumer^22,23,28^ (Fig. 4).

Also, a thin, elongate tubular structure was found within #IGKSC 324/19 (Fig. 3A_vi_) Based on its overall morphology, the structure was initially interpreted as a coprophagous tunnel produced by a small larva or annelid that penetrated the faeces from the surrounding sediment. However, this interpretation is unlikely. The extremely small diameter of the structure combined with its soft-tissue like morphology, closely bending along the internal surface (Fig. S11) of the coprolite wall is inconsistent with a burrow produced by an external coprophagous organism. Instead, these features suggests that the structure was incorporated into the coprolite, prior to its deposition.

This interpretation is further supported by the depositional environment. The surrounding matrix is devoid of bioturbation and contains framboidal pyrite, indicating dysoxic conditions and consequently, very limited activity of benthic invertebrate at the time of deposition.

We therefore consider the structure to most likely represent a piece of plant remain, such as a rhizoid or a comparable organ from an early vascular plant, already found at the locality^32^; which was ingested by the producer and not degraded during digestion.

The absence of any placoderm remains in the coprolites suggests the possible presence of multiple trophic niches in the Sula site palaeoecosystem. This may also be due to the fact that placoderms were not permanent inhabitants of this water body, or were not preyed upon by the producers. Also, there is the possibility that the predators regurgitated the placoderms skeletal elements. Only remains of *Bothriolepis*? sp. and a few fragmented dermal plates of the adult individuals of *Asterolepis radiata* were found in the top of the lens, directly beneath the basalts. The analysis of additional coprolite contents will help in confirming these hypotheses.

### Producer interpretation

As with any trace fossils, identifying the producer is a very complex, if not an impossible question to answer. The morphology and inner architecture of a coprolite can be used as proxies to constrain the identification of a potential producer^44^. Indeed a spiral or scroll valve in the posterior part of the intestine is a plesiomorphic feature shared amongst chondrichthyans (including acanthodians), non-teleostean actinopterygians, non-tetrapod sarcopterygians and placoderms^45–47^. Also, some early Palaeozoic jawless vertebrates may have possessed a scroll valve^48,49^.

Among the studied specimens, three coprolites – #IGKSC 324/19, #IGKSC 324/59A and #IGKSC 324/59B – display a spiral internal structure. Specimens #IGKSC 324/19 and #IGKSC 324/59A share a similar size and morphology, representing the first morphotype (Morphotype 1). In addition, specimens #IGKSC 324/25b, #IGKSC 324/31A and #IGKSC 324/31B, correspond to a second morphotype (Morphotype 2) and have a similar size and arrowhead morphology.

Every coprolite shows similar content, suggesting that their producers belonged to the same trophic levels. The diverse inclusions within the coprolites indicate an opportunistic feeding behaviour on prey from different trophic levels. This is best observed in specimen #IGKSC 324/59B (Fig. 3C), which contains remains of a porolepiform (tertiary consumer), a fleurantiid-like dipnoan (secondary consumer) a diplacanthid (secondary consumer) and a presumed acanthodid (primary consumer). Coprolites #IGKSC 324/19 and #IGKSC 324/59B were most likely produced by the apex predator of the locality as they contain faunal elements belonging to three trophic levels.

Based on their size, morphology and contents, the coprolites were likely produced by the following larger predators: *Glyptolepis* sp. (remains of which were also found within one coprolite), *Laccognathus* sp. and *Eusthenopteron* sp. The spiral inner architecture of specimens #IGKSC 324/19 and #IGKSC 324/59 allows us to narrow down their hypothetical producer. Although these taxa are fossil and incompletely anatomically preserved, phylogenetic bracketing can nonetheless help to refine taxonomical attribution to some of the producers. Indeed, out of the three aforementioned predators, the porolepiforms *Laccognathus* and *Glyptolepis* are most likely to possess spiral gut valves, as they are the sister group to dipnoans^50^, which are known to possess such an intestinal structure^45^. They are possible producers of the two spirally organised coprolites. On the contrary, *Eusthenopteron* belongs to a group more closely related to tetrapods, and may not have possessed a spiral valve. Thus, *Eusthenopteron* sp. was likely the producer of coprolites #IGKSC 324/31A and #IGKSC 324/31B, whereas *Laccognathus* sp. is the strongest candidate for specimens #IGKSC 324/19 and #IGKSC 324/59A.

### Comparison with contemporaneous assemblages

**Fig. 5:**
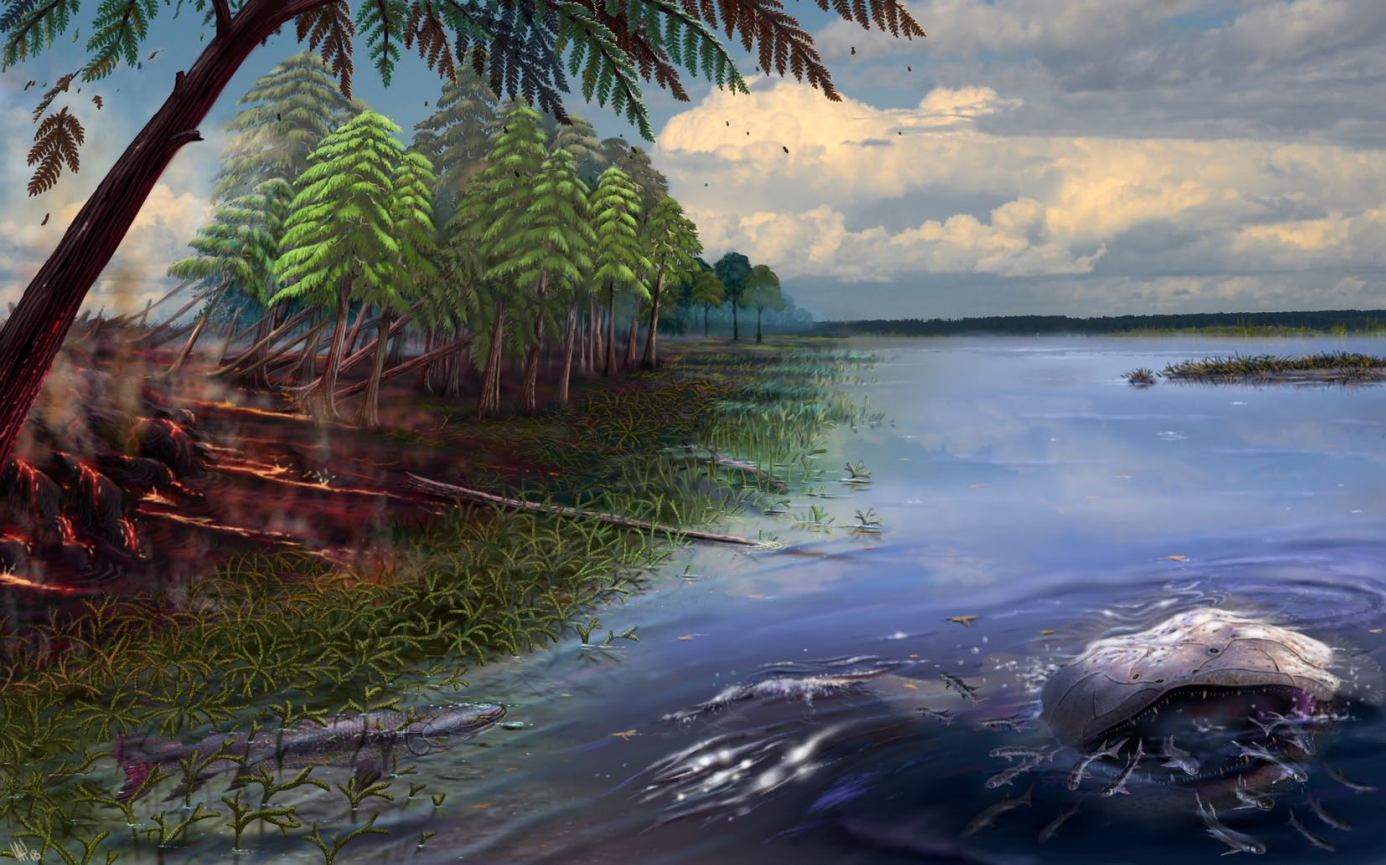
Artistic reconstruction of the final moments of life of the Sula site ecosystem. A *Laccognathus* hunts a school of acanthodians while *Eusthenopteron* hides in the *Helenia* thickets, as an advancing lava flow pushes onto an archaeopterid forest. Illustration by Michail Shekhanov, using the software “Corel Painter 2019” (https://www.painterartist.com/en/).

The vertebrate community of the Sula site, illustrated in Fig. 5, exhibits significant similarities with the contemporaneous fauna from the north-eastern sections of the Ust’-Chirka Formation (middle reaches of the Tsylma River, Middle Timan). Indeed, the latter yields one of the most diverse vertebrate assemblages within the Devonian sequence of the Timan Ridge^51^ and is interpreted as a low-salinity nearshore marine environment. In contrast, the Sula assemblage of the Kumushka Formation is entirely devoid of psammosteids, arthrodires, ptyctodontids, and actinopterygians. Nevertheless, it exhibits a much higher taxonomic diversity than the contemporaneous vertebrate communities recorded from the upper reaches of the Tsylma River, where coeval deposits formed under lacustrine and wetland conditions^51^.

The Sula assemblage is also quite similar to that of the contemporaneous non-marine Abava locality^52^. It is also similar to the marginal marine deposits of the Gauja Regional Stage (Baltic states and the Pskov Region of Russia )^53–55^ and more particularly to the Miguasha, Escuminac Formation^56,57^ (Canada).

The Miguasha assemblage^57–59^ is characterised by similar taxa found in the Sula site such as osteolepiforms (e.g. *Eusthenopteron foordi*), porolepiforms (e.g. *Holoptychius jarviki*), dipnoans (e.g. *Fleurantia denticulata*) and acanthodians (e.g. *Homalacanthus concinnus*). In addition to these taxa, the Miguasha assemblage contains an elpistostegid (*Elpistostege watsoni*), a coelacanth (*Miguashaia bureaui*), an actinopterygian (*Cheirolepis canadensis*) and several placoderms (e.g. *Bothriolepis canadensis*). This suggests that the Sula locality could have hosted an even more diversified faunal community.

Such a diverse assemblage of taxa, belonging to various trophic levels indicates a multi-level trophic web, providing evidence for the early establishment complex multi-level trophic chain at the Givetian-Frasnian transition. These results are consistent with Chevrinais, 2017^11^, who concluded that Devonian communities were structured across multiple trophic levels. Therefore, the Sula River locality offers a new crucial window into early non-marine vertebrate food webs.

## Methods

### Specimens

The studied material consists of four coprolite-bearing rock samples; #IGKSC 324/19 and #IGKSC 324/25b comprise one coprolite each, #IGKSC 324/31 contains 2 coprolites (#IGKSC 324/31A and #IGKSC 324/31B) and #IGKSC 324/59 contains three coprolites (#IGKSC 324/59A, #IGKSC 324/59B and #IGKSC 324/59C). The studied specimens are stored in the Chernov Geological Museum of the Institute of Geology, FRC Komi SC, UB of RAS (Syktyvkar), with collection number: IGKSC 324.

### Synchrotron microtomography and segmentation

The specimens studied in this project were scanned in 2018 by Paul Tafforeau at the beamline ID19 of the European Synchrotron Radiation Facility, Grenoble, France, using PPC-SRμCT (voxel size: 6.51 μm). The scans were made using white beam with a wiggler gap of 44 mm, filtered by 0.5 mm of tungsten and 5 mm of copper. The energy was 143 keV. The samples were placed at a propagation distance of 5 m from the detector. 5000 projections of 60 ms each were taken over 360 degrees. Tomographic slices were reconstructed using a filtered back-projection algorithm developed from the single distance phase retrieval^60,61^ using the software PyHST2^62^. Ring artefacts were corrected using an in-house correction tool^63^. Binned versions (bin2) were calculated to allow faster processing and screening of the samples since the full resolution data was large. The final volumes consist of stacks JPEG2000 images that were subsequently imported and segmented in the software Materialise Mimics 23.0. Standard threshold-based and manual segmentation was used to isolate individual inclusions appearing brighter than the surrounding coprolite matrix.

The resulting 3D meshes were imported in Materialise 3-matic 16.0 and rendered in the 3D software Blender 3.1.

### EDS analysis

For #IGKSC 324/59 and several additional unlabelled specimens, EDS analysis was performed using a TESCAN VEGA 3 LMH scanning electron microscope equipped with an Oxford Instruments X-Max 50 mm^2^ detector. The study was carried out at the Resource Center ‘Geoscience’ of the Institute of Geology, FRC Komi Scientific Center, UB of RAS (Syktyvkar, Russia) at an accelerating voltage of 20 kV, beam spot size of 180 nm, excitation region of up to 5 μm, acquisition time of ~ 600,000 counts, and vacuum of 0.02 Pa.

## Supplementary Information

Supplementary Information.

## Data Availability

The data reported in this paper are detailed in the main text and in the Supplementary Information. The raw CT data used can be accessed at: https://researchdata.se/en/catalogue/dataset/2026-13/1?previewToken=c4c86877-61ad-437c-b998-0a7be177f03b.
