# Supplementary material for: Investigating a Devonian vertebrate assemblage with synchrotron microtomography of coprolites
Source: Sci Rep. 2026 Aug 10;16:24728. doi: 10.1038/s41598-026-63718-2 (PMC13458357; doi:10.1038/s41598-026-63718-2)
Supplement: Supplementary file 1 [file 41598_2026_63718_MOESM1_ESM.docx]

**Supplementary information for**

Investigating a Devonian vertebrate assemblage with synchrotron microtomography of coprolites

Arnaud Rebillard*, Vincent Dupret, Pavel Beznosov, Per Erik Ahlberg

*Arnaud Rebillard

Email: [Arnaud.rebillard@mfn.berlin](mailto:Arnaud.rebillard@mfn.berlin)

**This PDF file includes:**

Fig. S1 to S11

Legends for Movies S1 to S4

**Other supporting materials for this manuscript include the following:**

Movies S1 to S4

Supp file 1

**Fig. S1:** Digital 3D rendering of coprolite #IGKSC 324/19 and its content (Sula fossil site, Nenets Autonomous Okrug, Arkhangelsk Region, RF, Kumushka Formation, Givetian-Frasnian transition beds). **A:** Sula2 sample coprolite modeled in 3D. **B:** tomogram displaying a transverse section along the plane shown in (**A**) showing the inclusions spirally organized. **C:** organization of the content in-situ; **C1:** porolepiform (incl holoptychiid) scale fragments. **C2–C5:** fleurantiid-like dipnoan. **C2:** elongated lepidotrichia. **C3:** short lepidotrichia. **C4:** lepidotrichia attached to a common base. **C5:** supraneural fragments. **C6:** possible poorly preserved dipnoan vertebra. **C7:** acanthodian scales. **C8:** vascular plant remain. Scale bars: 5 mm (A, K), 1 mm (B–J).

**Fig. S2:** Digital 3D rendering of coprolites #IGKSC 324/59A, #IGKSC 324/59B and #IGKSC 324/59C and their content (Sula fossil site, Nenets Autonomous Okrug, Arkhangelsk Region, RF, Kumushka Formation, Givetian-Frasnian transition beds). Coprolite A (**A**), coprolite B (**B**) and coprolite C (**C**). **D:** organization of the content in-situ in coprolite A **D1:** fleurantiid-like dipnoan prearticular toothplates. **D2:** dipnoan skull bones. **D3:** possible dipnoan shoulder girdle elements. **D4:** dipnoan cranial ribs. **D5:** dipnoan pleural rib**. D6:** osteichthyan scale fragments**. D7 :** diplacanthiform acanthodian fin spines **D8:** acanthodian fin spines. **D9:** acanthodid acanthodian scapulocoracoid. **D10:** acanthodian scales. **D11:** Dipnoan rib. **E** organization of the content in-situ in coprolite B. **E1:** holoptychiid porolepiform coronoid teeth. **E2:** holoptychiid porolepiform marginal teeth; **E3:** holoptychiid porolepiform parasymphysial tooth. **E4– E5:** fleurantiid-like dipnoan remains. **E4:** prearticular toothplate. **E5:** lepidotrichia. **E6:** osteichthyan scales. **E7:** sarcopterygian fangs **E8:** acanthodian scales; **F:** organization of the content in-situ in coprolite C; **F1:** possible dipnoan clavicle. **F2:** acanthodian scales. Scale bars: 5 mm (A, E, G, I), 1 mm (D, F, H)

**Fig. S3:** Digital 3D rendering of coprolite #IGKSC 324/25b (A) and its content (B) (Sula fossil site, Nenets Autonomous Okrug, Arkhangelsk Region, RF, Kumushka Formation, Givetian-Frasnian transition beds). **C:** fleurantiid-like dipnoan lepidotrichia. **D:** fleurantiid-like dipnoan tooth plate. **E:** dipnoan cranial rib. **F:** osteichthyan infraorbital bone. **G**: Possible acanthodian fin spine. Scale bars: 5 mm (A,B), 1 mm (C–H).

**Fig. S4:** Digital 3D rendering of coprolites #IGKSC 324/31A and #IGKSC 324/31B (A,D) and the organization of the content in situ (B,E) (Sula fossil site, Nenets Autonomous Okrug, Arkhangelsk Region, RF, Kumushka Formation, Givetian-Frasnian transition beds). **C1:** possible dipnoan unidentified element.. **C2:** possible dipnoan clavicle.. **C3:** osteichthyan lepidotrichia. **C4:** acanthodian scales. **F1:** fleurantiid-like dipnoan supraneural shafts. **F2:** fleurantiid-like dipnoan lepidotrichium. Scale bars: 0.5 cm (A–E), 1 mm (C,F).

**Fig. S5:** Digital 3D rendering of a fragmentary Eusthenopteron-like tristichopterid snout found within specimen #IGKSC 324/25b, but outside the coprolite (Sula fossil site, Nenets Autonomous Okrug, Arkhangelsk Region, RF, Kumushka Formation, Givetian-Frasnian transition beds); in lateral (**A,C**) and occlusal (**B,D**) views. **E:** cropped tomogram of the bone along the plane shown in (**A)**. Abbreviations**:** **f1:** fang1. **f2:** fang2. **f3:** fang3. **pm:** premaxilla. **vo:** vomer. Scale bars: 1 mm.

**Fig. S6:** Digital 3D rendering of a diplacanthiform acanthodian fin spine found in coprolite #IGKSC 324/59A (Sula fossil site, Nenets Autonomous Okrug, Arkhangelsk Region, RF, Kumushka Formation, Givetian-Frasnian transition beds) in lateral (**A**) and posterior (**B**) views. Scale bars: 1 mm.

**Fig. S7:** Digital 3D rendering of the fleurantiid-like dipnoan prearticular tooth plates found in coprolite #IGKSC 324/59A (Sula fossil site, Nenets Autonomous Okrug, Arkhangelsk Region, RF, Kumushka Formation, Givetian-Frasnian transition beds); in medial (**A1– C1**), lateral (**A2****– C2**) and occlusal (**A3– C3**) views. Scale bars: 1 mm

**Fig. S8:** Digital 3D rendering of the fleurantiid-like dipnoan tooth plates displaying radiating rows found in coprolite #IGKSC 324/59A (Sula fossil site, Nenets Autonomous Okrug, Arkhangelsk Region, RF, Kumushka Formation, Givetian-Frasnian transition beds), in occlusal view. Scale bars: 1 mm.

**Fig. S9:** Digital 3D rendering of the fleurantiidae-like dipnoan supraneural fragments found in coprolite #IGKSC 324/19 (Sula fossil site, Nenets Autonomous Okrug, Arkhangelsk Region, RF, Kumushka Formation, Givetian-Frasnian transition beds). **A,B,C:** supraneural fragments. **D,E:** supraneurals shaft. Scale bar: 1 mm.

**Fig. S10:** Digital 3D rendering of a preserved plant remain in coprolite #IGKSC 324/19 (Sula fossil site, Nenets Autonomous Okrug, Arkhangelsk Region, RF, Kumushka Formation, Givetian-Frasnian transition beds. Scale bars: 1 mm.

**Fig. S11:** Coprolite #IGKSC 324/19 in top view with (**A**) and without (**B**) the coprolitic matrix. This figure shows how the plant remain is closely bending along the internal surface.

Other supporting files:

**Movie S1:** Animation of coprolite #IGKSC 324/19, rotating about its vertical axis. The embedding coprolitic matrix disappears to reveal the three-dimensionally reconstructed inclusions. Scale bar: 5 mm

**Movie S2:** Animation of coprolites #IGKSC 324/59A, B and C, rotating about their vertical axis. The embedding coprolitic matrix disappears to reveal the three-dimensionally reconstructed inclusions. Note that acanthodian scales were not all segmented due to their high abundance (see main manuscript). Scale bar: 5 mm

**Movie S3:** Animation of coprolite #IGKSC 324/25b, rotating about its vertical axis. The embedding coprolitic matrix disappears to reveal the three-dimensionally reconstructed inclusions. Scale bar: 5 mm

**Movie S4:** Animation of coprolite #IGKSC 324/31A and B, rotating about their vertical axis. The embedding coprolitic matrix disappears to reveal the three-dimensionally reconstructed inclusions. Scale bar: 5 mm

**Supp file 1:** Powerpoint file including EDS results and corresponding SEM snapshots
